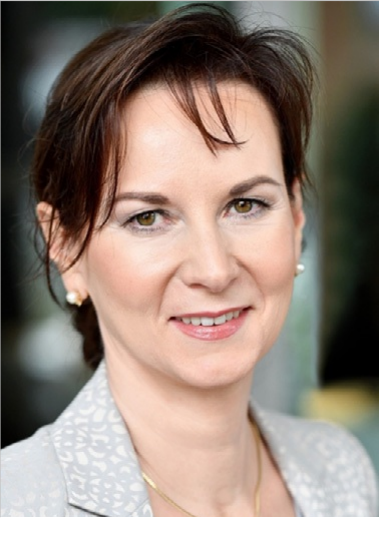# Catalytically Active Peptide–Gold Nanoparticle Conjugates: Prospecting for Artificial Enzymes

**DOI:** 10.1002/anie.201908625

**Published:** 2020-03-25

**Authors:** Dorian J. Mikolajczak, Allison A. Berger, Beate Koksch

**Affiliations:** ^1^ Department of Biology, Chemistry and Pharmacy Freie Universität Berlin Takustraße 3 14195 Berlin Germany

**Keywords:** artificial enzymes, catalysis, gold nanoparticles, peptides, self-assembly

## Abstract

The self‐assembly of peptides onto the surface of gold nanoparticles has emerged as a promising strategy towards the creation of artificial enzymes. The resulting high local peptide density surrounding the nanoparticle leads to cooperative and synergistic effects, which result in rate accelerations and distinct catalytic properties compared to the unconjugated peptide. This Minireview summarizes contributions to and progress made in the field of catalytically active peptide–gold nanoparticle conjugates. The origin of distinct properties, as well as potential applications, are also discussed.

## Graphical abstract

**The attachment of peptides** onto the surface of gold nanoparticles emerged as a promising strategy towards the creation of artificial enzymes. This Minireview summarizes the contributions to and progress made in the field of catalytically active peptide–gold nanoparticle conjugates over the past 15 years. The origin of distinct properties, as well as potential applications, are discussed.

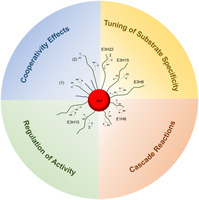

## 1 Introduction

Enzymes play a crucial role in accelerating virtually every organic transformation in nature, with unrivalled catalytic efficiency and specificity under mild conditions.^1^, ^2^ The superior catalytic performance of enzymes is directly related to their complex, folded protein structure, which accounts for the highly sophisticated enzyme–substrate interactions,^3^ but also for the fragility of the protein scaffold under harsh conditions.^4^, ^5^ To some extent, researchers have developed ways of circumventing such limitations, for example by the application of directed evolution.^6^, ^7^ Furthermore, for decades, the field of “biomimetic chemistry” and especially the subfield of “artificial enzymes”, has dealt with the translation of enzymatic properties into valid synthetic models^8^, ^9^ to develop catalysts that ideally possess rate constants and specificities similar to natural enzymes, while having greater stability under harsh conditions, and being reusable, easy to prepare and broadly applicable.^10^ Cyclodextrins,^8^, ^11^ polymers,^12^, ^13^, ^14^, ^15^ peptides,^16^, ^17^, ^18^, ^19^, ^20^, ^21^ and supramolecular systems^22^, ^23^, ^24^, ^25^, ^26^ have been used to this end. The field of nanotechnology has opened up a new avenue towards artificial enzymes based on metal nanoparticles (NPs).^27^, ^28^, ^29^ NPs possess a size‐regime similar to enzymes, a high surface‐area‐to‐volume ratio, thus a large number of potential catalytic sites, and the possibility for various surface modifications. In particular, gold nanoparticles (Au‐NPs) have received considerable attention due to their ease of surface functionalization and preparation, as well as biocompatibility,^30^, ^31^, ^32^ tunable stability,^32^ and exceptional optoelectronic^33^, ^34^ and inherent catalytic^27^, ^35^ properties. The surface of Au‐NPs can readily be functionalized with thiolated ligands that bind with high affinity to the Au surface through S−Au bonds.^36^ The desired ligands self‐assemble onto the Au surface generating functional monolayer‐protected gold clusters (Au‐MPCs) with well‐defined regions^37^, ^38^ and remarkable properties in molecular recognition^39^, ^40^ and catalysis.^41^, ^42^, ^43^, ^44^

In this context, peptide ligands further expanded the toolbox to functionalize Au‐NPs (Pep–Au‐NP= peptide–gold nanoparticle conjugate). Peptides are easy to access at low‐cost by solid‐phase peptide synthesis. Their design can be readily diversified by the choice of natural or unnatural amino acids, allowing for the fine‐tuning of reactivity and specificity of the ligand shell.^45^ The confinement of tailored peptides onto the surface of Au‐NPs creates dense peptide monolayers, in which novel hydrogen‐bond networks and charge‐relay interactions between individual functional groups are established. Thus, Pep–Au‐NPs are not only able to generate improved substrate and transition‐state interactions, and the associated rate enhancements, compared to unconjugated peptide, but can also execute novel catalytic mechanisms.

In this Minireview, we summarize progress made over the past 15 years in the field of catalytically active Pep–Au‐NPs, which is still in its youth.

We cover various topics, such as the origin of distinct properties evoked by the conjugation of peptides to the Au‐NPs and their similarities to natural enzymes, and provide insight into structure–function relationships of distinct conjugates. Ultimately the potential applications of these particles as artificial enzymes and heterogeneous catalysts for chemoenzymatic cascade transformations is highlighted.

## 2 The Origin of Distinct Catalytic Activities and Substrate Specificities

**Scheme 1:**
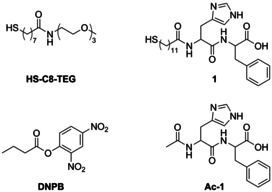
Structures of substrate, dipeptide, and thiol ligands used by Pengo et al.^46^ to obtain functionalized Au‐NPs and study esterase activity.

Pengo et al. pioneered the field of catalytically active Pep–Au‐NPs in 2005^46^ by reporting a simple dipeptidic model system synthesized by a ligand‐exchange protocol. Spherical precursor Au‐NPs capped by a stabilizing monolayer of a water‐soluble *N*‐(3,6,9‐trioxadecyl)‐8‐sulfanyloctanamide (**HS‐C8‐TEG**) were conjugated to a His‐Phe dipeptide **1** (Scheme 1) with an N‐terminal undecanethiol and an unmodified C‐terminus, resulting in Au@**1** (Scheme 1).

His is ubiquitous in the active sites of many esterases as its imidazole is able to perform either nucleophilic or general acid/base catalysis in tandem with other functional groups, such as carboxylic acids.^47^, ^48^ Hence, **1** fulfils the most basic requirements to function as an artificial esterase and to enable investigation of cooperativity effects between functional groups.^46^

Au@**1** and its corresponding sheerly peptidic N‐acetylated control variant, herein referred to as Ac‐**1,** were studied in their ability to cleave the ester substrate 2,4‐dinitrophenyl butyrate (DNPB, Scheme 1). At equal peptide concentrations, Au@**1** shows a rate acceleration of more than one order of magnitude over Ac‐**1** catalyzed DNPB hydrolysis at pH>7; at pH<7 rate acceleration of Au@**1** is even two orders of magnitude greater than that of Ac‐**1**.

**Figure 1:**
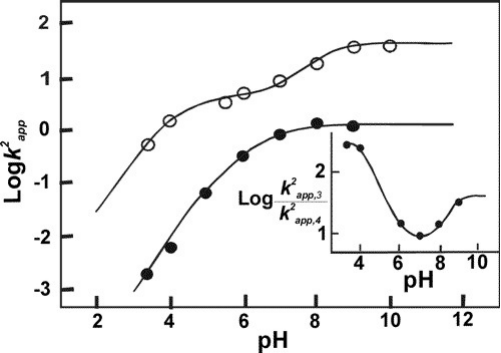
Dependence of Log of second‐order rate constant *k* ^2^ on pH for the Ac‐**1** (○) and and Au@**1** (•) catalyzed hydrolysis of DNPB. Inset showing the ratio of the second‐order rate constants on pH. Adapted with permission from ref. 46. Copyright 2005, American Chemical Society.

This rate acceleration arising solely from the NP‐conjugation of **1** was elucidated in further experiments by generating activity (Log of second‐order rate constant) versus pH profiles for Ac‐**1** and Au@**1** (Figure 1), to determine the nucleophilic species involved in catalysis.

Whereas the rate profile of Ac‐**1** indicates the presence of a single nucleophilic species with a p*K*_a_ of 6.6, which is assigned to the His imidazole, the profile of Au@**1** indicates the generation of two nucleophilic species, one having a p*K*_a_ of 4.2 (C‐terminal carboxylate) and the other 8.1 (His imidazole). The latter is 1.5 units higher than in unconjugated Ac‐**1** 46 and is a result of dipeptide immobilization on the Au‐NP. Since the attachment of peptides to the gold surface is directed by thiol–gold bonds, the outer most layer of Au‐**1** primarily consists of C‐terminal carboxylate anions. This high local negative charge density favours, by electrostatic interactions, the protonation of the proximal imidazole. Consequently, ester cleavage by commonly observed imidazole catalysis was ruled out and an alternative cooperative mechanism for ester hydrolysis was proposed. Thus, a C‐terminal carboxylate acts as a general base, deprotonating a surrounding water molecule and generating a nucleophilic hydroxide ion that initiates ester cleavage. The imidazolium ion described above assists in the catalytic process by stabilizing the negatively charged intermediate by hydrogen bonding, leading to the observed rate enhancements of Au@**1**.^46^

Rate improvements at the lower pH regimes are attributed to the carboxylate anion contributing to ester hydrolysis as an active nucleophile. There is no evidence that either of the newly proposed mechanisms, the cooperative carboxylate‐imidazole and the nucleophilic carboxylate are present in unconjugated peptides^46^

Despite Au@**1** showing only modest catalytic activity, the anchoring and confinement of amino acids or peptides onto the surface of Au‐NPs results in microenvironments that significantly affect the p*K*_a_ of functional groups and trigger new catalytic mechanisms. Such effects, made possible by preorganization and non‐covalent interactions, which only then enable specific catalytic mechanisms, is commonly observed in the active sites of folded enzymes.^49^, ^50^ The successful incorporation of such effects into model systems greatly improves our repertoire and understanding for the design of future enzyme mimics.

**Figure 2:**
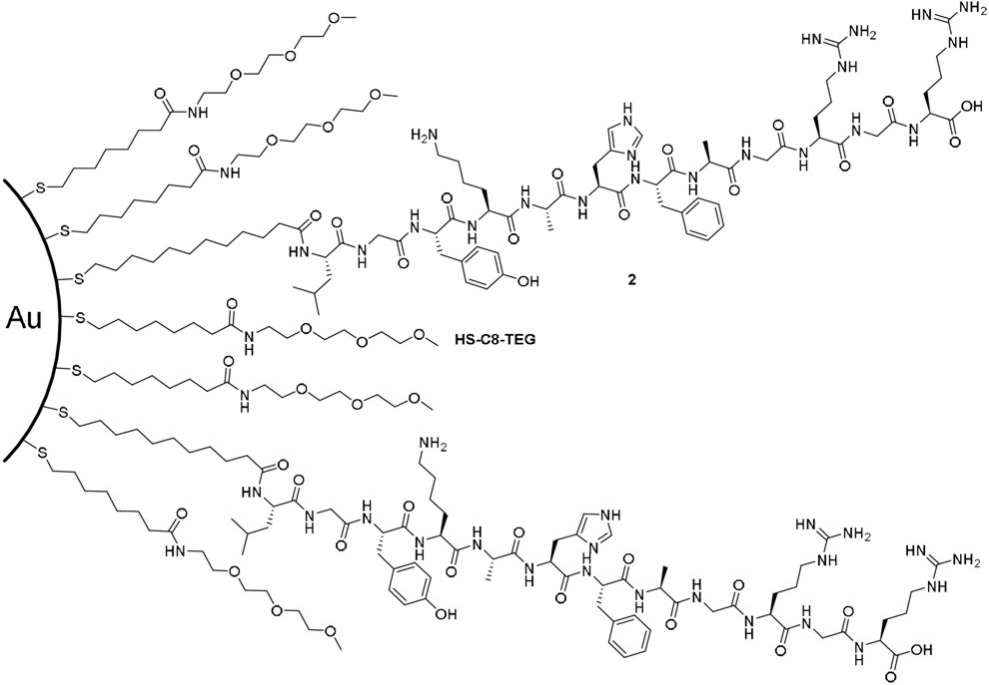
Schematic representation of Au@**2** composed of mixed monolayer of **HS‐C8‐TEG** and peptide **2** synthesized by Pengo et al. Drawn according to ref. 51.

In 2007, Pengo et al. extended the field of Pep–Au‐NP catalysts by reporting a more complex system.^51^ The dipeptide **1** of the previously reported conjugate was replaced with rationally designed peptide **2** consisting of 12 amino acids (Figure 2). Its design was based on a centrally located His functioning as the catalytic moiety by common imidazole catalysis. Thus, after immobilization onto the gold surface, His is buried inside the resulting monolayer, in a less solvated region, analogous to the active sites of natural enzymes. Lys and Arg were incorporated for the following reasons: i) to further stabilize the negatively charged transition state of ester hydrolysis, ii) together with His, to enable nucleophilic and/or general acid/base catalysis, and iii) to further stabilize the individual Pep–Au‐NPs due to electrostatic repulsion. Hydrophobic residues, such as Phe and Ala, were incorporated close to the His in order to enhance substrate binding and bring about proximity effects by hydrophobic interactions.^51^

**2** was conjugated to Au@**HS‐C8‐TEG**, analogous to Au@**1**, resulting in Au@**2** (originally referred to as Au‐PEP).^51^

Au@**2** and the S‐acetylated variant of **2** (Ac‐**2**) were studied as catalysts in the ester hydrolysis of DNPB. The results were compared to dipeptidic Au@**1**. At low pH, Au@**1** and Au@**2** behaved similarly, due to the nucleophilic carboxylate being active in both Pep–Au‐NP. However, Au@**2** exceeds the esterolytic efficiency of Au@**1** by almost one order of magnitude, up to pH 5. This corresponds to 3000‐ and 500‐fold rate accelerations compared to the unconjugated variants Ac‐**1** and Ac‐**2**, respectively, at pH 5, and a rate acceleration up to 40‐fold at pH>7 (Au@**2** vs. Ac‐**2**). The previously described cooperative carboxylate‐imidazolium mechanism evoked by the peptide monolayer was reported to be the most likely explanation for the rate enhancement.^51^

**Figure 3:**
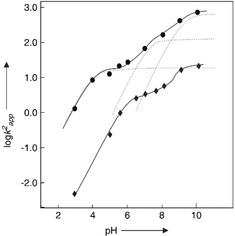
Dependence of logarithm of second‐order rate constant on pH for the Ac‐**2** (♦) and Au@**2** (•) catalyzed DNPB hydrolysis. Adapted with permission from ref. 52. Copyright 2007, WILEY‐VCH Verlag GmbH & Co. KGaA.

The activity versus pH plots of Ac‐**2** and Au@**2** were determined and compared (Figure 3).^51^, ^52^ A rather complex rate profile was obtained for Au@**2**, indicating three apparent p*K*_a_ values of 4.2 (C‐terminal carboxylate), 7.1 (His imidazole), and 9.9 (phenol group of Tyr). The overall higher catalytic activity of Au@**2** compared to Au@**1** was explained by the higher acidity of the protonated His imidazole, as indicated by the intrinsic p*K*_a_ being 0.9 units lower within the more complex peptide‐monolayer of Au@**2**. Despite His being buried inside the monolayer, it was still suggested that the carboxylate and the imidazole residues are spatially in close proximity, due to histidine showing a 1.1 unit higher p*K*_a_ than unconjugated Ac‐**2**.^51^

In further studies of Au@**2**, more complex 4‐nitrophenyl esters of Z‐protected amino acids leucine (Z‐Leu‐ONp) and glycine (Z‐Gly‐ONp) were applied in order to investigate the accommodation of substrates of different hydrophobicity within the peptide monolayer and their effect on the catalytic process. The studies indicated that Au@**2** shows different kinetics for the more hydrophobic substrate Z‐Leu‐ONp compared to Z‐Gly‐ONp and DNPB. For Z‐Leu‐ONp the formation of an intermediate, most likely the acetylated imidazole, was observed. The breakdown of the observed intermediate was thought to represent the new rate‐determining step of the catalytic process. However, up to a certain concentration, the cleavage of Z‐Gly‐ONp and Z‐Leu‐ONp by Au@**2** was shown to proceed with equal efficiency.^51^

Above that concentration, the nucleophilic attack of the imidazole becomes more efficient for Z‐Leu‐ONp. The increased nucleophilicity of the imidazole is explained by the accommodation of hydrophobic Z‐Leu‐ONp within the monolayer, leading to the exclusion of water and in turn increased hydrophobic and nucleophilic character in that region.^51^

The differences in accommodation and conversion of substrates with differing hydrophobicities strongly hint at more potential hidden within the design of the peptide monolayer. Exploiting differences in solvation, as well as changing the spatial proximity between peptide chains and varying the flexibility of the peptide scaffold, are potential parameters to fine‐tune catalytic performance.

After this pioneering work of Pengo et al.,^46^, ^51^ the field remained silent for a decade until in 2017 we resumed studies on Pep–Au‐NP catalysts. Compared to the aforementioned works in which mixed monolayers of **HS‐C8‐TEG** and peptide‐derived ligands were applied, our approach was based on Au‐NPs exclusively coated with peptides. To that end, Cys‐containing peptides were used as stabilizing ligands to obtain spherical Pep–Au‐NPs.^53^ As a result, higher peptide concentrations at the Au surface and higher activities are expected. However, since Pengo et al.^46^, ^51^ did not provide absolute values for their peptide loadings, quantitative comparisons between their and our catalytic rates cannot be drawn. Another caveat is that, in general, care should be taken in comparing the absolute values of kinetic parameters of Pep–Au‐NPs because catalytic activity depends on the size, shape, and peptide loading of individual Pep–Au‐NP batches.

Regarding kinetic assays, in addition to the pseudo‐first‐order reaction kinetic determinations of the kind carried out by Pengo et al.,^46^, ^51^ we also carried out saturation kinetic experiments. Thus, turnover number *k*_cat_ and Michaelis–Menten constant *K*_M_ were able to be calculated, giving access to catalytic efficiency *k*_cat_/*K*_M_ at maximum catalyst velocity. Improved catalytic efficiencies (originating from increases in *k*_cat_ and decreases in *K*_M_) for Pep–Au‐NPs, compared to the unconjugated peptide variants, were observed in all studies, in agreement with the works of Pengo et al.^46^, ^51^ These can be attributed to two cooperative phenomena: first, the cooperative carboxylate‐imidazole mechanism; and second, complex peptide–peptide and/or peptide–substrate interactions that take place within the peptide monolayer and assist in the catalytic process by facilitating substrate binding and stabilization. However, the exact nature and extent of the latter effect has yet to be elucidated.

In an attempt to gain further insight into the properties of Pep–Au‐NPs, and to tie in with the background given by Pengo et al.,^46^, ^51^ we studied the effect the location of the catalytic center within the peptide monolayer has on esterolytic activity and substrate specificity.^53^

**Figure 4:**
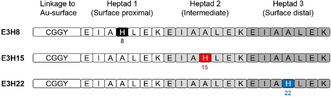
Peptide sequences of the E3HX series including **E3H8**, **E3H15**, and **E3H22** and positions of histidine. Adapted with permission from ref. 53. Copyright 2018, WILEY‐VCH Verlag GmbH & Co. KGaA.

To this end, we designed a series of three peptides (E3HX‐series) that differ only in the position of the catalytic center, and thus, after conjugation to the Au‐NPs, the region within the peptide monolayer at which catalysis takes place. The peptides consist of three repetitions of seven amino acids (Heptads 1–3) to ensure identical chemical environments around the catalytic centers. Between two Glu residues present in each heptad repeat, a His was incorporated as the catalytically active moiety in order to mimic esterase activity and trigger imidazole‐carboxylate interactions after conjugation to the Au‐NPs. His was sequentially moved along the heptad repeats to give peptides **E3H8**, **E3H15**, and **E3H22** (Figure 4). Incorporation of an N‐terminal cysteine enabled covalent thiol–gold bonding.

The three peptides were directly conjugated to Au‐NPs to give Au@**E3H8**, Au@**E3H15**, and Au@**E3H22**. In the following, we refer to the regions within the peptide monolayer at which catalysis takes place as surface proximal, intermediate and surface distal for Au@**E3H8**, Au@**E3H15,** and Au@**E3H22**, respectively. Comparison among this series was possible as size, shape and peptide loading between the batches were similar.^53^

In order to assess the substrate specificity of the three defined regions of the peptide monolayer for substrates of different hydrophobicities, model substrate 4‐nitrophenyl acetate (4‐NPA/*p*NPA) and more sophisticated Z‐protected *p*‐nitrophenyl esters of amino acids Glu, Ala, Phe, and Leu (in ascending hydrophobicity order) were employed. For the latter only pseudo‐first‐order reactions were performed owing to the poor solubility of the substrates in aqueous media.^53^

**Figure 5:**
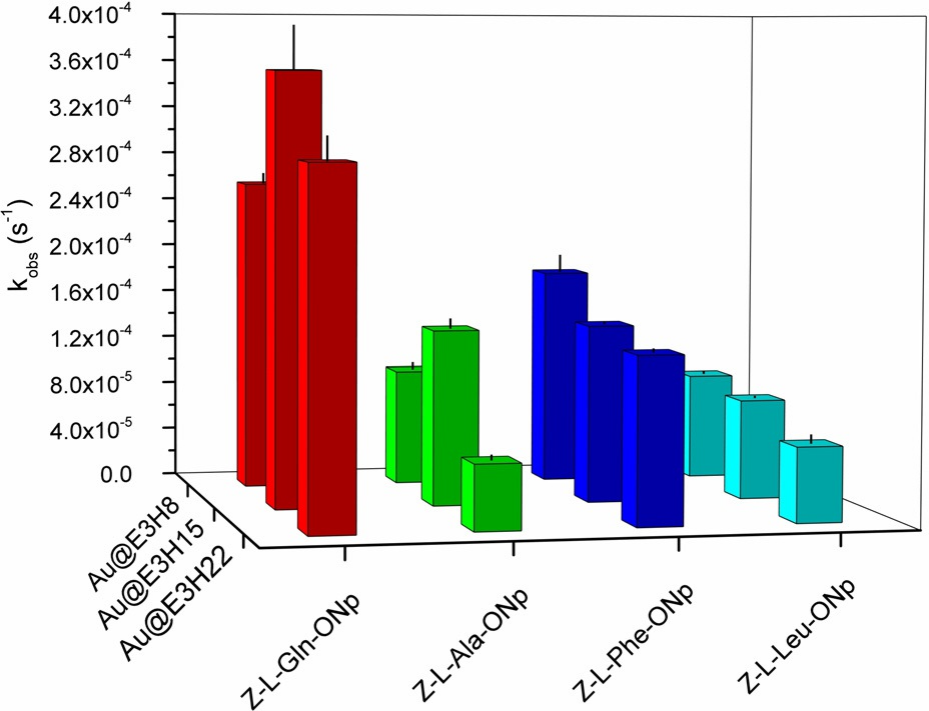
Pseudo‐first‐order rate constants for the hydrolysis of different 4‐nitrophenyl esters. Adapted with permission from ref. 53. Copyright 2018, WILEY‐VCH Verlag GmbH & Co. KGaA.

As shown in Figure 5, the highly hydrophobic substrates Z‐l‐Phe‐ONp and Z‐l‐Leu‐ONp were most efficiently cleaved by Au@**E3H8**. The increased rate of ester hydrolysis for Z‐l‐Phe‐ONp and Z‐l‐Leu‐ONp by Au@**E3H8** can be explained by taking into account the surrounding amino acids flanking the active surface‐proximal His. The flanking residues comprise the non‐polar amino acids Ala, Leu, and Ile. For Au@**E3H8**, these amino acids are in less solvated states, since regions within the peptide monolayer and associated amino acids closer to the nanoparticle surface are usually less solvated, due to the high density of the confined peptide chains.^54^ This results in an increased overall hydrophobic character of the surface proximal region that can stabilize more hydrophobic substrates; therefore, even though the overall amount of water, necessary for the process of ester hydrolysis is lower, relatively higher rates of reaction compared to the intermediate and surface distal regions are achieved.^53^

In contrast, the more hydrophilic substrates 4‐NPA, Z‐l‐Gln‐ONp and Z‐l‐Ala‐ONp were cleaved most rapidly by Au@**E3H15** in the intermediate region of the peptide monolayer. To explain that, we compared the environments of the three different regions of the peptide monolayer in terms of solvation of functional groups, rigidity/flexibility of the peptide chains, and cooperativity in substrate binding. The latter was assessed by determining the Hill coefficients (*n*) of the obtained sigmoidal saturation curves for 4‐NPA hydrolysis.^55^ Based on these values, it was assumed that cooperativity in substrate binding for the other somewhat less hydrophobic substrates Z‐l‐Gln‐ONp and Z‐l‐Ala‐ONp follows the same trend, namely, falls off with increasing distance from the NP.^53^

While the surface proximal region of Au@**E3H8** shows the greatest amount of cooperativity in substrate binding (*n=*3.39), the low water content leads to a slower overall catalytic process as hydrophobic interactions do not play a key role for the more hydrophilic substrates. For the highly flexible and solvent‐exposed surface distal region of Au@**E3H22** cooperativity was lowest (*n=*2.48), presumably due to the high flexibility of the peptide chains, leading to a lower effective peptide density. However, the highly water‐exposed surface distal region speeds up the process of ester hydrolysis, resulting in a higher rate of reaction compared to the surface proximal region. We concluded that the intermediate region of the peptide monolayer shows a more optimal balance between solvation of functional groups, rigidity of the peptide scaffold and cooperativity (*n=*2.65), which might result in catalytic environments, analogous to natural enzymes, with improved hydrophilic ester substrate cleavage.^53^

This set of experiments verified that the catalytic properties of Pep–Au‐NPs can not only be tuned by design of the desired active site but, equally importantly, by considering the region at which catalysis takes place and the properties of the secondary coordination sphere that are generated within the peptide monolayer. This leads to a broad and complex set of design rules, and, consequently, an expanded pallet of potential catalysts with distinct properties. In general, further studies are necessary in order to fully understand and rationalize these design principles.

## 3 Structure–Function Relationships

A major advantage of applying peptides as ligands for the functionalization of Au‐NPs is their ability to adopt secondary, tertiary, or even quaternary structures. With this in mind, we were particularly interested in the structure–function relationships of Pep–Au‐NPs. We distinguish between two types of systems: first, systems in which peptides are designed to possess no secondary structure propensity but intrinsically organize within the peptide monolayer, which leads to catalytic activity; and second, systems in which peptides are designed to form defined secondary structures at the NP surface, thus, providing a defined arrangement of functional groups that only then results in catalytic activity.

For the first system, we studied how disruption of the intrinsic peptide arrangement surrounding the nanoparticles impacts catalysis. To this end, two peptides based on the well‐characterized parallel, heterodimeric IAAL‐E3/IAAL‐K3 coiled‐coil system^56^ were chosen and synthesized.^57^ The two peptides, namely esterolytically active **E3H15** and its catalytically inactive complementary peptide **K3** are specifically designed such that one peptide alone does not display a defined secondary structure. Physical mixing of the two specifically designed peptides in aqueous media induces formation of α‐helices that wrap around each other to form stable α‐helical coiled‐coil bundles.^58^ **E3H15** was immobilized onto the surface of Au‐NPs as described above to give spherical Au@**E3H15**.

**Figure 6:**
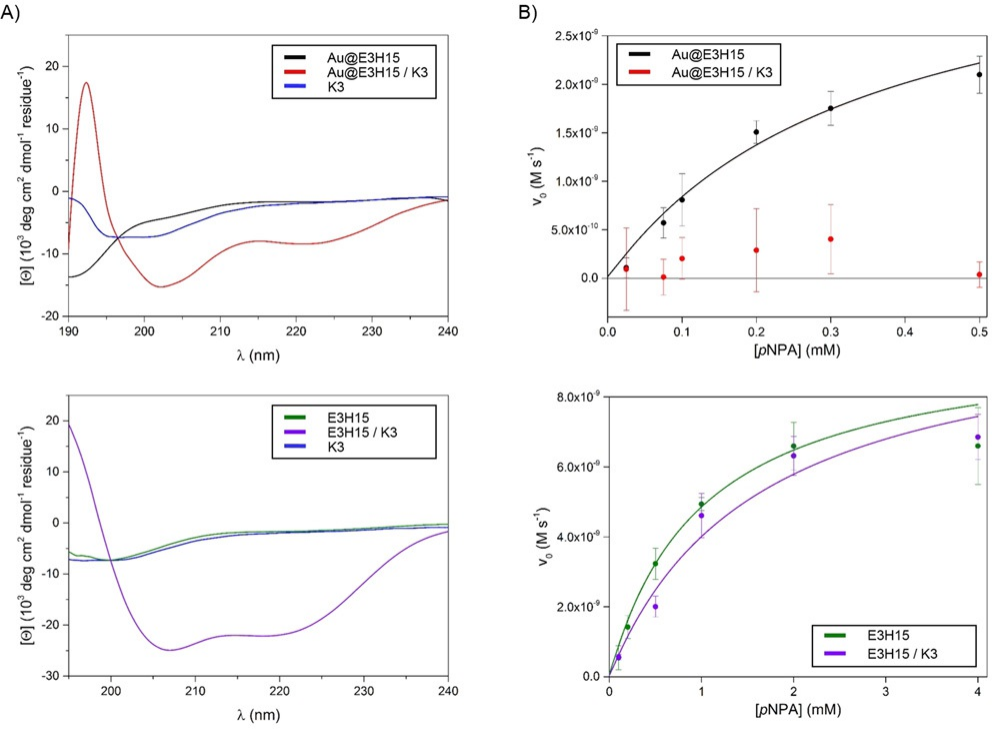
A) Normalized CD spectra of **E3H15** and Au@**E3H15** in the absence or presence of **K3**. B) Saturation curve of 4‐NPA hydrolysis catalyzed by **E3H15** and Au@**E3H15** in the absence or presence of **K3**. Adapted with permission from ref. 57. Copyright 2018, WILEY‐VCH Verlag GmbH & Co. KGaA.

We then studied the effect of changing peptide conformation on the esterolytic activity of Au@**E3H15** and **E3H15** by the addition of equimolar **K3**. Circular dichroism (CD) spectroscopy revealed no defined secondary structure for the individual peptides of Au@**E3H15**, **E3H15**, and **K3** by themselves (Figure 6 A). However, the mixing of **K3** with Au@**E3H15** and **E3H15**, respectively, results in the formation of α‐helical structures, as indicated by the appearance of two new CD minima at 222 and 202 nm. Owing to the peptide‐design principle, coiled‐coil structures were adopted.^57^

The kinetic results revealed an overall 85 % decrease in catalytic efficiency for coiled‐coils comprised of Au@**E3H15**/**K3**, whereas only a slight decrease in catalytic efficiency was observed for the unconjugated coiled‐coil variant of **E3H15** in the presence of **K3** (Figure 6 B).^57^

The significant reduction of catalytic efficiency of conformationally changed Au@**E3H15**, induced by interactions with **K3**, were attributed to a combination of effects. The presence of Lys‐rich **K3** within the Glu‐rich **E3H15** monolayer can lead to changes in p*K*_a_ values, as the complementary positive charges of the Lys residues counteract any p*K*_a_ effects evoked by the negatively charged Glu residues of **E3H15**. Hence, the cooperative imidazolium‐carboxylate mechanism is diminished and rate accelerations are decreased. Furthermore, after the change in conformation, the peptides become involved in defined, more rigid α‐helical coiled‐coil structures. The flexibility of functional groups to form intermolecular binding cavities or access beneficial orientations for substrate binding is hindered. Also, within coiled‐coil structures, the position and orientation of individual amino acid side chains is predefined. Thus, possible intramolecular interactions of particular residues necessary for catalysis to occur, such as His and Glu, are decreased. In any of the described cases, the formation of α‐helical coiled‐coil structures on the surface of Au‐NPs results in lower rate accelerations.^57^

Even though not apparent from the CD‐spectroscopic data, peptides apparently do possess internal organization within the monolayer, leading to a conformation in which functional groups are judiciously placed, and the perturbation of which results in significant losses in activity. Riccardi et al. reported that even less complex Au‐MPCs consisting of oligo(ethylene glycol) are capable of forming transient cavities in which small organic molecules are accommodated.^40^ It is therefore not out of the question that peptides composed of various different functional groups are able to form similar clefts to beneficially accommodate substrate molecules and subsequently convert them with higher efficiency. However, further experiments on Pep–Au‐NPs are necessary in order to test this hypothesis.

We also directed our research efforts towards investigating the second Pep–Au‐NP system mentioned above, in which the formation of secondary structures leads to catalytic activity instead of diminishing it. Simultaneously, we wanted to prove that Pep–Au‐NPs are able to catalyze chemical reactions other than ester hydrolysis. Therefore, we designed a Pep–Au‐NP system that mimics the activity of the zinc‐containing metalloenzyme carbonic anhydrase (CA).^59^ We extracted the design rules for Pep–Au‐NPs obtained from the E3HX series and utilized the brush‐like nature of the peptide monolayer to design a peptide that, after conjugation to Au‐NPs, develops a minimalistic version of the active site of CA, instead of mimicking only the catalytic center. The peptide design is based around the amyloid‐forming IHIHIQI (IHQ) peptide by Rufo et al.^60^ The IHQ peptide assembles into parallel β‐sheet structures, where His in positions *i* and *i*+2 form an interstrand 3‐His–Zn^II^‐binding site. In the presence of Zn^II^, the peptide cleaves ester substrate 4‐NPA by a Zn^II^‐bound hydroxide mechanism. The IHQ peptide was further modified (referred to as IHQ‐NP) to include a Cys‐containing linker sequence for Au‐NP conjugation and a hydrophilic region at the surface distal region; the latter should assist the catalytic process by facilitating product release or proton/solvent shuttle processes, analogous to the active site of CA.^61^

**Figure 7:**
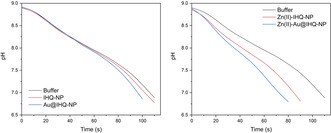
CO_2_‐hydration activity of IHQ‐NP and Au@IHQ‐NP monitored by the delta‐pH method in (left) absence and (right) presence of ZnCl_2_ according to ref. 59.

CD spectroscopy revealed that IHQ‐NP assembles into β‐sheet structures, indicated by a single ellipticity minimum around 215 nm, also when conjugated to Au‐NPs. Thus, the prerequisite for the 3‐His–Zn^II^ center is fulfilled.^59^ Au@IHQ‐NP and its unconjugated variant IHQ‐NP marginally catalyze the hydration of CO_2_ to HCO_3_^−^ in Tris‐buffered solution, in the absence of Zn^II^ (Figure 7). The addition of Zn^II^ significantly increases rates of CO_2_ hydration by 20 % and 50 % for IHQ‐NP and Au@IHQ‐NP, respectively, compared to the buffer control. More interestingly, Au@IHQ‐NP catalyzes the hydration of CO_2_ with superior rates compared to the unconjugated peptide. Thus, rate acceleration due to the development of a peptide monolayer is not only limited to self‐assembled systems with an internal peptide organization, but is extendable also to Pep–Au‐NPs that form defined secondary structures and execute catalytic mechanisms by developed metal centers.^59^ These results also strongly indicate that the catalytic rate enhancement of Pep–Au‐NPs is not merely a consequence of a change in reaction mechanism, but is an effect evoked by a more intricate set of interaction events that occur within the monolayer. Understanding these effects at the molecular level also proves beneficial for the design of Pep–Au‐NPs for other applications in molecular recognition or binding of a target molecule/receptor.

## 4 Cascade Transformations

Pep–Au‐NPs are hybrid inorganic–organic nanomaterials in which the inorganic Au‐NP is usually used as a template to immobilize catalytically active peptides onto the metal surface, as previously mentioned. However, a plethora of publications report Au‐NPs as catalysts performing a variety of hydrogenation reactions^62^, ^63^ or oxidative transformations^64^, ^65^, ^66^, ^67^, ^68^ at the Au surface. Consequently, Pep–Au‐NPs are potential candidates to function as cascade catalysts, performing two sequential transformations in aqueous media under mild conditions in one pot.

**Scheme 2:**
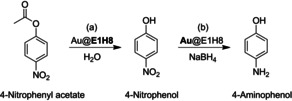
a) Au@**E1H8**‐catalyzed hydrolysis of 4‐NPA to 4‐NP and b) following NaBH_4_‐mediated reduction of 4‐NP to 4‐AP. Bold‐face type indicates the catalytically relevant component. Adapted with permission from ref. 69. Copyright 2018, WILEY‐VCH Verlag GmbH & Co. KGaA.

To provide a proof‐of‐principle that Pep–Au‐NPs are indeed suitable catalysts for cascade transformations, we opted to study the ester cleavage of model substrate 4‐NPA to obtain 4‐nitrophenol (4‐NP) using an appropriately designed peptide monolayer (Scheme 2). Then, without work‐up, in one pot, 4‐NP functions as the new reactant in a subsequent NaBH_4_‐mediated hydrogenation reaction taking place at the Au‐NP surface to yield 4‐aminophenol (4‐AP).^69^

The peptide monolayer, mimicking esterase activity, was accessed by truncation of the aforementioned esterolytically active peptide series E3HX. The resulting sequence consisting of 11 amino acids (**E1H8**) still includes the basic principles of a charge‐relay network that is able to accelerate ester hydrolysis. **E1H8** was covalently conjugated by performing the NaBH_4_‐mediated reduction reaction of HAuCl_4_ to obtain spherical Au@**E1H8**.

**Figure 8:**
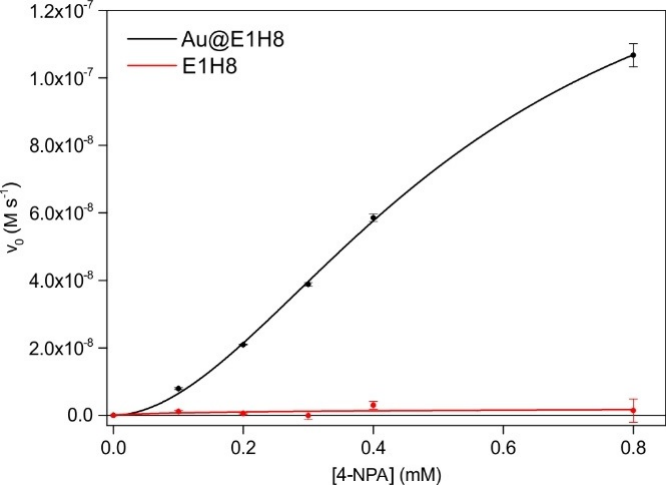
Hill plot of **E1H8**‐ and Au@**E1H8**‐catalyzed hydrolysis of 4‐NPA. Adapted with permission from ref. 69. Copyright 2018, WILEY‐VCH Verlag GmbH & Co. KGaA.

The ester hydrolysis of 4‐NPA was investigated and it was shown that Au@**E1H8** functions as an efficient esterase mimic (Figure 8), comparing favorably to esterase mimics in the literature.^60^, ^70^, ^71^, ^72^, ^73^, ^74^, ^75^ Even though smaller in peptide chain length compared to the E3HX series, the saturation curve still showed sigmoidal behavior, indicating cooperativity in substrate binding between individual peptide chains.^69^ Direct comparison of kinetic parameters between Au@**E1H8** and the Au@E3HX series is not possible as peptide loading significantly differs.

**Figure 9:**
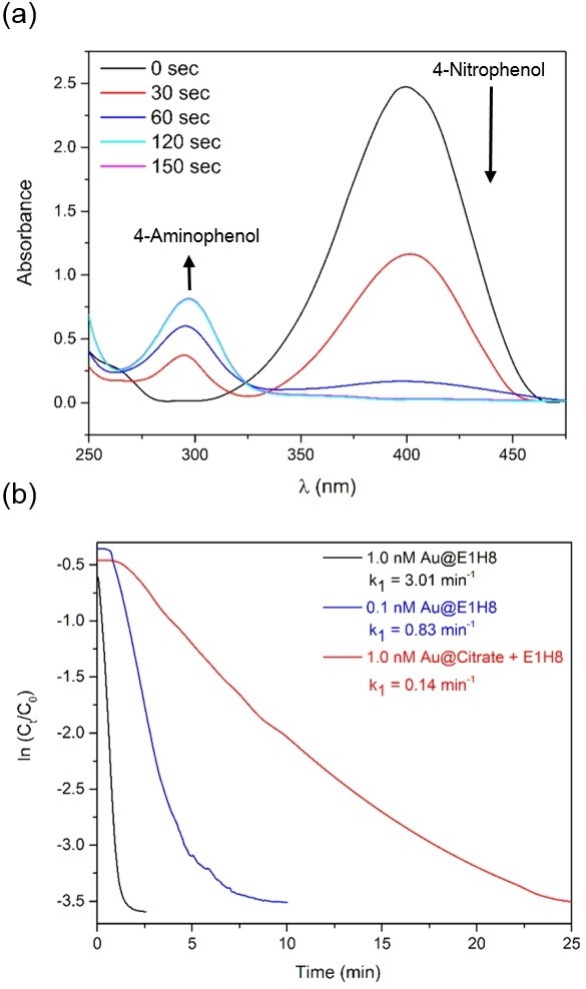
a) Time‐dependent UV/Vis spectra and b) plot of ln(*C*_t_/*C*_0_) (*C*_0_= initial substrate concentration and *C*_t_= substrate concentration at time t) versus reaction time for the reduction of 4‐NP to AP catalyzed by Au@**E1H8** and Au@Citrate mixed with unconjugated **E1H8** and respective k_1_. Adapted with permission from ref. 69. Copyright 2018, WILEY‐VCH Verlag GmbH & Co. KGaA.

Following 4‐NPA hydrolysis, excess NaBH_4_ was added to the reaction mixture, initiating the hydrogenation reaction of 4‐NP to 4‐AP at the Au‐NP surface (Figure 9). Reactions run with citrate‐capped Au‐NPs (Au@Citrate) in the presence of unconjugated **E1H8** were performed as a control. The observed pseudo‐first order rate constants (*k*_1,obs_) for Au@**E1H8** exceed the control by more than 20‐fold as well as literature reported Au‐NPs capped by cetyltrimethylammonium bromide (CTAB), citrate, mixed layers of citrate/CTAB or citrate/polyvinylpyrrolidone.^76^ The **E1H8**‐peptide monolayer is able to significantly contribute to the overall catalytic process taking place at the Au surface. We explain the contributions of the peptide to the gold catalyzed hydrogenation reaction accordingly: the **E1H8** monolayer beneficially interacts with the 4‐NP through hydrogen bonds and/or electrostatic interactions; 4‐NP is retained and the residence time in the vicinity of the Au surface is increased; the 4‐NP therefore accumulates within the monolayer, and is brought into close proximity to the gold surface, which in turn accelerates the catalytic process.^69^ However, detailed mechanistic studies regarding the surface processes that contribute to catalysis are necessary in order to clarify the complex interactions that take place within and around Pep–Au‐NPs.

## 5 Summary and Outlook

The pioneering works of Pengo et al. reviewed here demonstrated that novel molecular mechanisms can be achieved by the immobilization of peptides on gold‐NP surfaces, namely, a hitherto unknown carboxylate‐imidazole‐mediated ester hydrolysis and that the substrate can modulate the activity of such catalysts based on polarity/hydrophobicity effects. Our subsequent studies on the E3HX series showed that catalytic efficiency correlates both with the position of the catalytic center, and with the hydrophobicity of the substrate. We then investigated structure–activity relationships in the context of either an α‐helical coiled‐coil or a β‐sheet conformation and found, in the former, that disruption of the intrinsic peptide arrangement reduces catalytic efficiency, and, in the latter, that strand association within the monolayer enables the formation of the catalytically competent 3‐His–Zn^II^ center. Finally, a water‐compatible, one‐pot approach involving an immobilized truncated version of the E3HX series, as well as the Au surface itself, was shown to carry out sequential cascade ester hydrolysis and hydrogenation with good efficiency.

Although this field of research is still in a relatively early stage, the works reviewed here have provided information about the key aspects of these complex systems. Overall, new researchers entering this field must consider the type of reaction(s) to be catalyzed on which substrate(s), which residues are needed to accomplish the chemistry, whether secondary structure is needed, for instance, if metal coordination plays a role, where to place the catalytic center with respect to the particle surface, and the judicious placement of formal charges and hydrophobic groups (also with a view to providing stability).

All things considered, these systems still do not enable the high levels of catalytic efficiency achieved by natural enzymes. Looking forward, it will be important to more carefully engineer the secondary coordination sphere. Based on the work reviewed here, the parameters to be optimized are apparent. Rather than the kind of studies in which a peptide sequence is rationally designed and then conjugated to the nanoparticle, it would seem prudent to adopt approaches inspired by combinatorial chemistry and directed protein evolution because the influence of conjugation is not easily predicted. The efforts spent in learning will be rewarded by the development of new enzyme‐like catalysts that hold promise for applications in new technologies.

## Conflict of interest

The authors declare no conflict of interest.

## Biographical Information

*Dorian Jamal Mikolajczak studied chemistry at Humboldt Universität zu Berlin (B.Sc) and Freie Universität Berlin (M.Sc.). In 2016 he joined the group of Beate Koksch at the Freie Universität Berlin as a Ph.D. candidate. His research interests include the design and synthesis of new catalytic systems*.

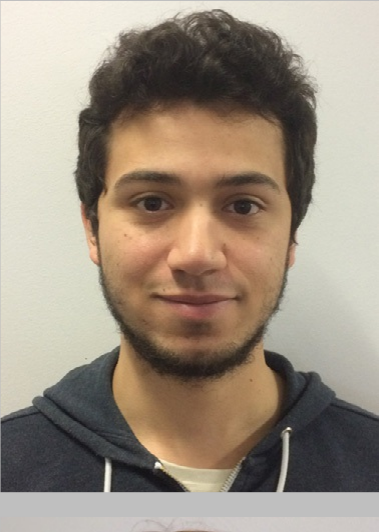

## Biographical Information

*Allison Ann Berger received a B.A. degree from Reed College and a Ph.D. degree from TSRI La Jolla*, *both in Chemistry. After completing a two‐year postdoctoral stay at the Max‐Planck‐Institute for Infection Biology as an Alexander von Humboldt fellow (2005–2007), she joined the group of Beate Koksch as staff scientist*.

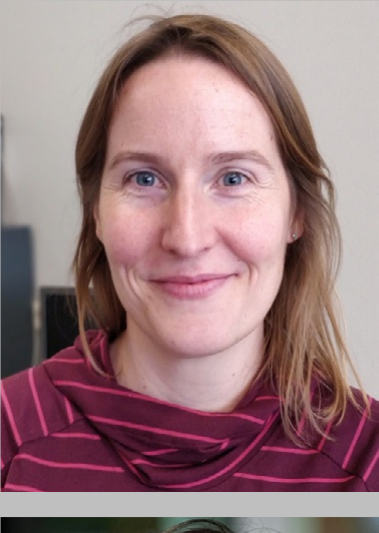

## Biographical Information

*Beate Koksch received a Ph.D. degree from University Leipzig and pursued postdoctoral studies at TSRI La Jolla and a postdoctoral lecture qualification at University Leipzig with Klaus Burger. She has been Professor of Chemistry at Freie Universität Berlin since 2004. Her group investigates fluorinated amino acids in the context of peptides and proteins, studies complex folding mechanisms in neurodegenerative diseases, and develops new multivalent scaffolds also for catalysis*.